# Reduced fitness and abnormal cardiopulmonary responses to maximal exercise testing in children and young adults with sickle cell anemia

**DOI:** 10.14814/phy2.12338

**Published:** 2015-04-06

**Authors:** Robert I Liem, Madhuri Reddy, Stephanie A Pelligra, Adrienne P Savant, Bo Fernhall, Mark Rodeghier, Alexis A Thompson

**Affiliations:** 1 Department of Pediatrics, Hematology, Oncology & Stem Cell Transplant, Ann & Robert H. Lurie Children's Hospital of Chicago, Northwestern University Feinberg School of MedicineChicago, Illinois, USA; 2 Department of Pediatrics, Pulmonary Medicine, Ann & Robert H. Lurie Children's Hospital of Chicago, Northwestern University Feinberg School of MedicineChicago, Illinois, USA; 3 Department of Kinesiology and Nutrition, University of Illinois at ChicagoChicago, IL, USA; 4 Rodeghier ConsultantsChicago, Illinois, USA

**Keywords:** Cardiopulmonary fitness, exercise testing, sickle cell

## Abstract

Physiologic contributors to reduced exercise capacity in individuals with sickle cell anemia (SCA) are not well understood. The objective of this study was to characterize the cardiopulmonary response to maximal cardiopulmonary exercise testing (CPET) and determine factors associated with reduced exercise capacity among children and young adults with SCA. A cross-sectional cohort of 60 children and young adults (mean 15.1 ± 3.4 years) with hemoglobin SS or S/β^0^ thalassemia and 30 matched controls (mean 14.6 ± 3.5 years) without SCA or sickle cell trait underwent maximal CPET by a graded, symptom-limited cycle ergometry protocol with breath-by-breath, gas exchange analysis. Compared to controls without SCA, subjects with SCA demonstrated significantly lower peak VO_2_ (26.9 ± 6.9 vs. 37.0 ± 9.2 mL/kg/min, *P *<* *0.001). Subjects demonstrated slower oxygen uptake (ΔVO_2_/ΔWR, 9 ± 2 vs. 12 ± 2 mL/min/watt, *P *<* *0.001) and lower oxygen pulse (ΔVO_2_/ΔHR, 12 ± 4 vs. 20 ± 7 mL/beat, *P *<* *0.001) as well as reduced oxygen uptake efficiency (Δ*V*_E_/ΔVO_2_, 42 ± 8 vs. 32 ± 5, *P *<* *0.001) and ventilation efficiency (Δ*V*_E_/ΔVCO_2_, 30.3 ± 3.7 vs. 27.3 ± 2.5, *P *<* *0.001) during CPET. Peak VO_2_ remained significantly lower in subjects with SCA after adjusting for age, sex, body mass index (BMI), and hemoglobin, which were independent predictors of peak VO_2_ for subjects with SCA. In the largest study to date using maximal CPET in SCA, we demonstrate that children and young adults with SCA have reduced exercise capacity attributable to factors independent of anemia. Complex derangements in gas exchange and oxygen uptake during maximal exercise are common in this population.

## Introduction

Sickle cell anemia (SCA) is a common inherited blood disorder associated with abnormal hemoglobin production and characterized by chronic hemolysis, a proinflammatory state and endothelial dysfunction (Solovey et al. 1997; Reiter et al. 2002; Hebbel et al. 2004; Brittain and Parise 2007). Classic manifestations include, but are not limited to, moderate to severe anemia, acute pain episodes, acute lung injury, and varying degrees of cardiopulmonary disease that develops with increasing age. As such, the disease impact on physical function among affected individuals is significant. Both adults and children with SCA, either directly or by proxy, consistently report poor physical functioning on health-related quality of life surveys (Panepinto et al. 2005). Six-minute walk distances, used frequently as an indirect measure of exercise capacity, are reduced in children and adults with SCA (Sachdev et al. 2011; van Beers et al. 2014). Direct assessments of aerobic exercise capacity by maximal cardiopulmonary exercise testing (CPET), however, are rarely performed for clinical indications in this population.

Pathophysiologic factors such as chronic anemia and cardiopulmonary disease, even when subclinical, may contribute to reduced exercise capacity among individuals with SCA. In their study of 17 adult women with SCA undergoing maximal CPET, Callahan et al. found that exercise limitation was most frequently associated with altered gas exchange responses during exercise, suggestive of pulmonary vascular disease (Callahan et al. 2002). Early studies in children with SCA undergoing CPET have also found physiologic derangements associated with reduced exercise capacity, ranging from inadequate cardiac compensation for anemia during exercise to exaggerated ventilatory responses attributed to increased physiologic dead space ventilation (Alpert et al. 1981; Covitz et al. 1983; Pianosi et al. 1991a,b).

Studies to date that have examined exercise capacity in SCA by maximal CPET are limited by small sample sizes, variability in measurements reported, and lack of appropriate comparison groups. The full spectrum of physiologic exercise responses associated with reduced exercise capacity in individuals with SCA therefore remains poorly understood. Furthermore, the safety of routinely conducting maximal CPET in this population has not been established. The objective of this study was to characterize the cardiopulmonary response to maximal exercise on CPET with breath-by-breath gas exchange and to evaluate potential contributors to reduced exercise capacity among children and young adults with SCA. We hypothesize that reduced exercise capacity in this population is associated with significant derangements in the cardiopulmonary response to exercise, including oxygen uptake, oxygen pulse, and ventilatory efficiency.

## Materials and Methods

This was a prospective, cross-sectional study of 60 subjects with SCA and 30 controls without SCA enrolled at Ann & Robert H. Lurie Children's Hospital of Chicago (formerly Children's Memorial Hospital until June 2012) from July 2010 to March 2014. Lurie Children's Hospital is a 288-bed, urban teaching hospital affiliated with the Department of Pediatrics at Northwestern University Feinberg School of Medicine. The Institutional Review Board approved this study, and all participants underwent informed consent/assent according to institutional standards prior to participation.

### Participant characteristics

All 60 subjects with SCA were followed in the Comprehensive Sickle Cell Program at Lurie Children's Hospital and met the following inclusion criteria: (1) SCA defined as either hemoglobin SS or S/β^0^ thalassemia confirmed by hemoglobin electrophoresis and (2) 8–21 years of age. Subjects on hydroxyurea therapy were included but those enrolled on a chronic, monthly transfusion protocol were excluded due to the disease-modifying effects of chronic transfusions in SCA. Potential subjects were also excluded for the following: (1) motor deficits or neurologic injury, (2) symptomatic avascular necrosis, or (3) history of syncope, dizziness, or chest pain during exertion. For a 2:1 ratio, we recruited 30 controls without SCA or sickle cell trait matched by race, sex, and age within 2 years of subjects. Controls were recruited from subject sibling pools or through advertisements placed in clinic settings throughout the hospital.

### Cardiopulmonary exercise testing

All subjects and controls underwent maximal CPET following a modified Godfrey protocol (Godfrey et al. 1971) using an electronically braked VIAsprint 150p Ergometric Bicycle (Carefusion Corp., San Diego, CA) with pediatric configurations. For subjects, CPET was performed at least 2 weeks from any vaso-occlusive pain episode requiring hospitalization and at least 3 months from any blood transfusion. We performed a screening evaluation on the day of testing to ensure subjects had no symptoms that would preclude them from performing maximal CPET. This modified Godfrey cycle ergometry protocol was a graded, symptom-limited test with simultaneous collection of breath-by-breath, gas exchange data using a Vmax Encore 29C metabolic cart (CareFusion Corp., San Diego, CA). Initial workload and workload increments were based on height of each subject: 10 watts for height <120 cm, 15 watts for 120–150 cm, and 20 watts for >150 cm. Work increments by continuous ramping of work load occurred at 1-min intervals. A cadence of 60 revolutions per minute was maintained with each increase in workload until the subject reached maximal effort, defined as a respiratory exchange ratio (RER) of 1.1 or higher, or stopped due to volitional exhaustion and inability to maintain cadence. Continuous pulse oximetry and electrocardiography (ECG) occurred throughout the warm-up, exercise and recovery phases. Oxygen saturation (i.e., SpO_2_ values) from pulse oximetry was recorded at baseline and every minute during exercise and recovery. Abnormal SpO_2_ and SpO_2_ response were defined as ≤ 95% at either baseline or peak exercise, or a significant drop in value from baseline to peak exercise, respectively.

### Outcome measures and definitions

Our primary outcome measure was peak oxygen consumption (VO_2_), the reference standard for measuring exercise capacity by maximal CPET. Peak VO_2_ was calculated from 20-sec averages of data points and defined as the highest weight-adjusted value obtained in the last minute of exercise prior to the recovery phase. Secondary outcomes included the following CPET parameters: (1) total exercise time; (2) work rate (WR); (3) minute ventilation (V_E_), oxygen consumption (VO_2_) and carbon dioxide production (VCO_2_); (4) breathing reserve (BR), calculated as *V*_E_/maximum voluntary ventilation (MVV); (5) peak heart rate (HR) and HR reserve, calculated as the difference between peak HR during exercise and baseline HR; (6) ventilatory threshold (VT), measured by a single individual using the V-slope method; and (7) slopes representing ΔVO_2_/ΔWR, Δ*V*_E_/ΔVCO_2_, ΔVO_2_/ΔHR, and Δ*V*_E_/ΔVO_2_. Where appropriate, gas exchange values and slopes were calculated from 10-sec averages of data points obtained on breath-by-breath analysis. Standard demographic data were collected as well as baseline hemoglobin on the day of CPET. To monitor safety of testing, phone calls were made to all subjects at 1 and 2 weeks following CPET to document adverse events.

### Statistical considerations

Standard descriptive analysis was performed and the normality of data distribution was assessed. Categorical data were evaluated using Pearson's chi-square test and Fisher's exact test where appropriate. Continuous data were analyzed using Student t-tests with the Mann–Whitney-Wilcoxon test used for variables with unequal variance or a nonnormal distribution. Linear regression was performed for the entire group of subjects and controls and for subjects, separately, using weight-adjusted peak VO_2_ as the dependent variable and age, sex, body mass index (BMI), hydroxyurea use, baseline hemoglobin, and white blood cell (WBC) count as covariates. All analyses were performed using IBM SPSS Statistics (Version 20, Chicago, IL, IBM).

## Results

### Participant characteristics and CPET safety

A total of 60 subjects with SCA and matched 30 controls without SCA or sickle cell trait successfully completed CPET during the study period. There was no significant difference in age or distribution of sex between the two groups, although subjects with SCA had lower BMI (21 ± 4 vs. 24 ± 7 kg/m^2^, *P *=* *0.013) (Table1). Baseline Hb (8.7 ± 1.3 vs. 12.9 ± 1.3 g/dL, *P *<* *0.001) and total WBC count (9.6 ± 3.3 vs. 6.0 ± 1.9 × 10^3^, *P *<* *0.001) were significantly lower and higher, respectively, in subjects with SCA when compared with controls. Hydroxyurea use was reported by 23/60 (38%) of subjects with SCA.

**Table 1:** Demographic Information for Subjects and Controls

Variable	Subjects with SCA				Controls without SCA				*P* value*
	*N*=	Mean (SD)	Median	IQR	*N*=	Mean (SD)	Median	IQR	
Age (years)	60	15.1 (3.4)	15.0	4.8	30	14.6 (3.5)	14.0	5.0	0.55
BMI (kg/cm^2^)	60	21 (4)	20	5	30	24 (7)	24	7	0.01
Hemoglobin (g/dL)	60	8.7 (1.3)	8.5	2.1	28	12.9 (1.3)	13.1	2.1	<0.001
WBC count (×10^3)^	60	9.6 (3.3)	10.1	4.1	28	6.0 (1.9)	5.6	3.3	<0.001
Test Time (min)	60	5.6 (1.3)	5.6	1.9	30	7.8 (2.0)	7.9	2.7	<0.001
Respiratory Exchange Ratio	60	1.3 (0.1)	1.3	0.2	30	1.2 (0.1)	1.1	0.2	<0.001
Peak VO_2_ (mL/min/kg)	60	26.9 (6.9)	25.9	9.7	30	37.0 (9.2)	34.6	14.7	<0.001
Peak Work Rate (watts)	60	108 (37)	107	59	30	153 (49)	154	58	<0.001
Peak Heart Rate (bpm)	58	177 (12)	176	21	30	180 (13)	188	21	0.32
Heart Rate Reserve (bpm)	58	99 (14)	100	22	30	109 (15)	109	23	0.005
Peak Minute Ventilation (L/min)	60	60.2 (20.3)	59.9	23.5	30	78.5 (21.4)	76.0	22.1	<0.001
Breathing Reserve (breaths/min)	57	72 (18)	72	21	22	76 (27)	79	26	0.36
Ventilatory Threshold (mL/min)	60	1.0 (0.3)	1.0	0.4	30	1.3 (0.3)	1.3	0.4	<0.001
Percent peak VO_2_ at VT (%)	60	68 (10)	69	15	30	58 (10)	57	15	<0.001
Δ*V*_E_/ΔVCO_2_	60	30.3 (3.7)	30	4.9	30	27.3 (2.5)	26.9	2.6	<0.001
ΔVO_2_/ΔWork Rate (mL/min/watt)	60	9.2 (2.1)	9.0	2.2	30	11.6 (2.1)	11.3	3.2	<0.001
ΔVO_2_/ΔHeart Rate (mL/beat)	60	11.7 (3.7)	12.0	5.6	30	20.3 (6.8)	20.1	9.4	<0.001
Δ*V*_E_/ΔVO_2_	60	41.9 (8.1)	41.5	13.7	30	31.9 (4.7)	30.6	4.7	<0.001

* Significant at *P *<* *0.05.

All 60 (100%) subjects with SCA and 22/30 (73%) controls met criteria for a maximal test, defined as an RER ≥ 1.1, and in all, testing was terminated due to excessive participant fatigue. In 10/60 (17%) subjects, but no controls, SpO_2_ was ≤95% at either baseline or peak exercise, or SpO_2_ significantly dropped from baseline to peak exercise. No serious adverse event occurred during or up to 2 weeks after CPET in any subject or control. Only 1/60 (2%) subjects reported vaso-occlusive pain during a weeklong dance camp after her study visit. She required hospitalization for her pain 13 days after CPET, but the episode was determined to be unrelated to study participation.

### Cardiopulmonary responses to maximal exercise testing

Nearly all the major indicators of CPET performance and gas exchange were adversely affected in subjects with SCA when compared with controls without SCA. The removal of controls who did not meet criterion for a maximal test from the analysis did not significantly alter our results (data not shown). On average, total exercise time was significantly lower in subjects with SCA when compared with controls without SCA (5.6 ± 1.3 vs. 7.8 ± 2.0 min, *P *<* *0.001). Subjects achieved a significantly lower peak WR (108 ± 37 vs. 153 ± 49 watts, *P *<* *0.001) and weight-adjusted peak VO_2_ (26.9 ± 6.9 vs. 37.0 ± 9.2 mL/kg/min, *P *<* *0.001) during maximal exercise. Ventilatory threshold was similarly reduced in subjects with SCA compared with controls (1.0 ± 0.3 vs. 1.3 ± 0.03 L/min, *P *<* *0.001). Although we found no significant difference in peak HR achieved between the two groups during exercise, HR reserve was significantly lower in subjects with SCA (99 ± 14 vs. 109 ± 15 bpm, *P *=* *0.005).

**Figure 1:**
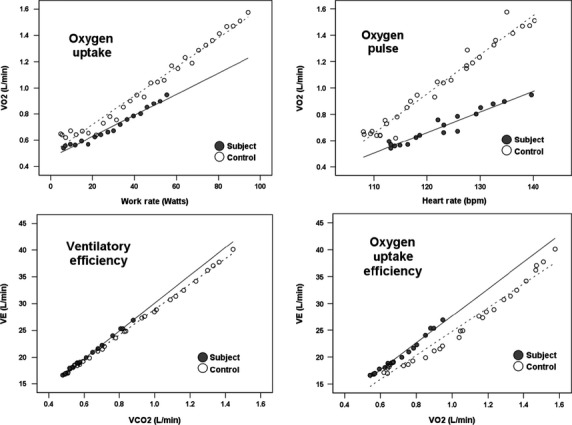
Differences in slopes for ΔVO_2_/ΔWR (upper left), ΔVO_2_/ΔHR (upper right), Δ*V*_E_/ΔVCO_2_ (lower left) and Δ*V*_E_/ΔVO_2_ (lower right) calculated from 10-sec averaged data from the first 3 and 5 min for subjects with SCA and controls without SCA, respectively. Cutoff values for data points were based on the minimal test time that allowed for inclusion of the same number of data points for all subjects and controls.

Slopes calculated from breath-by-breath data collected during CPET indicated significant differences in gas exchange as well as oxygen uptake and delivery between subjects with SCA and controls without SCA (Fig.1). When compared with controls without SCA, subjects with SCA demonstrated slower oxygen uptake and reduced oxygen uptake efficiency, as evidenced by lower ΔVO_2_/ΔWR (9 ± 2 vs. 12 ± 2 mL/min/watt, *P *<* *0.001) and higher Δ*V*_E_/ΔVO_2_ (42 ± 8 vs. 32 ± 5, *P *<* *0.001), respectively. Subjects with SCA demonstrated lower ΔVO_2_/ΔHR (12 ± 4 vs. 20 ± 7 mL/beat, *P *<* *0.001), indicating lower oxygen pulse during exercise. Ventilatory efficiency was also reduced, as evidenced by higher Δ*V*_E_/ΔVCO_2_ (30.3 ± 3.7 vs. 27.3 ± 2.5, *P *<* *0.001). Among subjects, only ΔVO_2_/ΔWR was significantly lower in subjects who demonstrated an abnormal SpO_2_ or SpO_2_ response when compared with subjects who did not (data not shown).

We also compared weight-adjusted peak VO_2_ in subjects with SCA who did or did not demonstrate an abnormal cardiopulmonary response to CPET. Among subjects with SCA, 25/60 (42%) met criteria for an abnormal cardiopulmonary response to exercise testing, defined as having a HR reserve or ΔVO_2_/ΔWR at least two standard deviations below the mean for controls without SCA or Δ*V*_E_/ΔVCO_2_ at least two standard deviations above the mean for controls without SCA. When compared with subjects without an abnormal cardiopulmonary response to CPET, subjects with an abnormal response demonstrated lower peak VO_2_ (25.1 ± 6.4 vs. 28.1 ± 7.0 mL/kg/min, *P *=* *0.2), although the finding was not statistically significant.

### Clinical predictors of cardiopulmonary fitness

Multivariate regression models were derived to determine independent clinical predictors of peak VO_2_ in subjects with SCA and controls without SCA. We found that in a model that included both subjects and controls, peak VO_2_ remained significantly lower in subjects with SCA even after adjusting for age, sex, BMI, baseline hemoglobin, and total WBC count (Table2). In this combined model, subject type, age, sex, BMI, and baseline hemoglobin were independent predictors of peak VO_2_. Whether or not criteria for a max were achieved did not significantly alter the results of the model.

**Table 2:** Predictors of peak VO_2_ in subjects with SCA and controls without SCA

Variable	β Coefficient	Standard error	*P* value*
Controls without SCA	5.93	2.06	0.005
Age (years)	−0.84	0.18	<0.001
Female Sex	−6.95	1.06	<0.001
BMI (kg/m^2^)	−0.63	0.12	<0.001
Baseline hemoglobin (g/dL)	1.30	0.43	0.003
Baseline WBC (×10^3)^	−0.28	0.18	0.12

* Significant at *P *<* *0.05.

In a separate model that included only subjects with SCA, we found that age, sex, BMI, and baseline hemoglobin remained independent predictors of peak VO_2_ whereas total WBC count and hydroxyurea use were not significantly associated with peak VO_2_ (Table3). Specifically, we found that higher age, male sex, greater BMI, and higher baseline hemoglobin were significantly associated with higher peak VO_2_. This model explained 52% of the variability associated with peak VO_2_ in our subjects with SCA.

**Table 3:** Predictors of peak VO_2_ in subjects with SCA only

Variable	β Coefficient	Standard error	*P* value*
Age (years)	−0.90	0.21	<0.001
Female Sex	−6.48	1.28	<0.001
BMI (kg/m^2^)	−0.49	0.19	0.013
Baseline hemoglobin (g/dL)	1.38	0.55	0.016
Baseline WBC (×10^3)^	−0.29	0.21	0.17
Hydroxyurea Use	−0.98	1.40	0.487

* Significant at *P *<* *0.05.

## Discussion

This study demonstrated several important findings that strengthen our understanding of cardiopulmonary fitness and exercise responses among children and young adults with SCA. We showed that CPET at maximal exertion is safe and did not directly cause vasoocclusive pain either during or after testing. We found that exercise capacity, defined by peak VO_2_, is significantly lower in children and young adults with SCA when compared with that measured in matched controls without SCA or sickle cell trait. Reduced exercise capacity was also associated with numerous derangements in the cardiopulmonary response to exercise challenge in our subjects, including reduced efficiency in oxygen uptake and delivery as well as ventilation. Among subjects with SCA, age, sex, BMI, and baseline hemoglobin were independent predictors of weight-adjusted peak VO_2_.

Our results are aligned with those from several other studies that have indicated impairment of physical functioning and exercise capacity in children and adults with SCA. However, the majority of these studies have relied on either self-reported outcomes of physical function on health-related quality of life assessments or surrogates and estimates of exercise capacity such as 6-min walk distance and extrapolation from submaximal exercise testing, respectively (Charache et al. 1983; McConnell et al. 1989; Hackney et al. 1997; Barst et al. 2010; Gordeuk et al. 2011; Sachdev et al. 2011; Waltz et al. 2013). Fewer recent studies have utilized maximal CPET, considered the gold standard for measuring cardiopulmonary fitness (Callahan et al. 2002; Anthi et al. 2007; Das et al. 2008; Chaudry et al. 2013; van Beers et al. 2014). In our study, RER values calculated at the end of exercise testing indicated subjects with SCA reached maximal effort, with termination of testing from excessive fatigue rather than poor effort. Importantly, our study supports the safety of adopting maximal CPET with gas exchange analysis for assessing cardiopulmonary fitness and physiologic responses to exercise in this population. The ability to safely use maximal CPET for accurate assessment of peak VO_2_ in SCA is necessary given that cardiopulmonary fitness, defined by peak VO_2_, represents one of the most important predictors of morbidity and all-cause mortality in the general population (Blair et al. 1996; Wei et al. 1999; Myers et al. 2002; Carnethon et al. 2005).

The CPET slope data derived from breath-by-breath, gas exchange analysis augment our understanding of peak VO_2_ by emphasizing those specific determinants of the cardiovascular and ventilatory responses to exercise, which may be useful even when peak VO_2_ cannot be achieved in individuals with SCA or other conditions (Cooper et al. 2014). Our most important findings by gas exchange include evidence for slower and less efficient oxygen uptake, lower oxygen pulse and a reduction in ventilatory efficiency among subjects with SCA undergoing CPET. Some of these abnormalities in gas exchange have previously been reported in patients with SCA whereas others have not. For example, abnormal cardiovascular responses to maximal exercise, indicated by lower peak HR and reduced cardiac output, have been observed in children with SCA (Alpert et al. 1981; Covitz et al. 1983). However, the impact of cardiovascular disease on oxygen transport and delivery, reflected by slower oxygen uptake (ΔVO_2_/ΔWR) and lower oxygen pulse (ΔVO_2_/ΔHR), has not previously been reported in children with SCA. Similarly, an exaggerated ventilatory response to exercise resulting in a higher Δ*V*_E_/ΔVCO_2_ has previously been seen in children and adults with SCA, presumably due to dead space ventilation (Miller et al. 1973; Pianosi et al. 1991b; Callahan et al. 2002). Our study, however, also demonstrates the potential negative impact of physiologic dead space on oxygen uptake efficiency (Δ*V*_E_/ΔVO_2_), which has recently been shown to be lower among adults with SCA undergoing submaximal exercise testing (Charlot et al. 2014).

Despite these results, we cannot implicate a single process that is primarily responsible for limiting peak VO_2_ in our subjects with SCA given the normal integration of the metabolic, cardiovascular and ventilatory responses to exercise. That an abnormal cardiopulmonary response to exercise is present among subjects with SCA is not surprising as SCA is associated with an increased risk for developing chronic lung injury and cardiac dysfunction with age. Several known complications of SCA, including pulmonary vascular disease and diastolic dysfunction (Sachdev et al. 2011), could explain the gas exchange patterns observed in our subjects through compromises in pulmonary blood flow (i.e. dead space ventilation) or cardiac output during exercise challenge. The relationship between abnormal cardiopulmonary responses and reduced exercise capacity is supported by the 10% reduction in peak VO_2_ we observed in subjects who demonstrated an abnormal cardiopulmonary response during CPET. Alternatively, limitations in oxygen transport during exercise could in part be explained by peripheral blood flow restriction from increased red blood cell sickling in our subjects.

We also explored clinical contributors to peak VO_2_ in our study population. Not surprisingly, higher age, male sex, higher BMI, and higher hemoglobin were all independently associated with higher peak VO_2_ in participants with and without SCA. The impact of sex on cardiopulmonary fitness in children has been well described and may be independent of other factors known to affect peak VO_2_ (Rutenfranz et al. 1982; Janz et al. 1998). The developmental aspects of peak VO_2_ are also known to track with physical growth, pubertal maturation and increases in skeletal mass with age (Davies et al. 1972; Krahenbuhl et al. 1985; Armstrong et al. 1991). Given the oxygen carrying capacity of hemoglobin, it is not surprising that hemoglobin is also an independent contributor to peak VO_2_ in SCA. Correction of anemia is associated with improvements in exercise capacity in children and adults with end-stage renal disease and congestive heart failure (Metra et al. 1991; Martin et al. 1993; Mancini et al. 2003). However, these clinical variables explain just over 50% of the variability in peak VO_2_ in our subjects with SCA. In our combined regression model, SCA remained an independent predictor of lower peak VO_2_ after adjustment for age, sex, BMI, baseline hemoglobin and baseline WBC count, thus indicating other physiologic variables or pathophysiologic processes may contribute to exercise limitation in this population.

There are some limitations to our study and results. Our cross-sectional sample population, though moderate in size, limited the number of variables we could examine as predictors of peak VO_2_ in our multivariable models. However, it is comparable to sample sizes in exercise physiology studies of similar detail and in fact, comprises the largest reported group of children with the most severe forms of sickle cell disease (i.e., hemoglobin SS and S/β^0^ thalassemia) undergoing maximal CPET. We did not repeat exercise testing in any of our subjects to evaluate the reproducibility of our primary CPET measurements. Additionally, our primary outcome of peak VO_2_ represents a less rigorous measure of maximal cardiopulmonary fitness when compared with VO_2_ max, defined as a plateau in VO_2_ consumption despite an increase in work load. However, safety concerns precluded the use of a supramaximal protocol to try to confirm VO_2_ max in our subjects with SCA. Moreover, few children achieve a true VO_2_ max, which may be equivalent to peak VO_2_ in children despite the addition of supramaximal testing (Barker et al. 2011). We also did not include findings from standard cardiopulmonary evaluations, such as echocardiography or pulmonary function testing, in our models. However, our primary objective was to evaluate the dynamic cardiopulmonary responses to exercise challenge, which may be independent of findings on static assessments of cardiopulmonary function. Although potentially useful, the invasiveness and challenges associated with directly measuring cardiac output or physiologic dead space during exercise precluded our performing these procedures in this study. Finally, that we found no significant association between hydroxyurea use and peak VO_2_ in our subjects with SCA may in part reflect our inability to adjust for adherence to therapy in those subjects taking the medication.

In summary, maximal CPET is safe in children and young adults with SCA, suggesting that acute exercise challenge is well tolerated in this population even at high levels of exercise intensity and physical exertion. When compared with their peers, children and young adults with SCA demonstrate significantly reduced fitness levels. Reduced exercise capacity in SCA may be attributed to complex derangements in the cardiopulmonary and metabolic responses to exercise that are independent of chronic anemia. Our findings highlight the need to further elucidate the impact of reduced exercise capacity on disease severity and clinical outcomes in SCA. A clearer understanding of the disease-modifying potential of regular exercise will guide the development of evidence-based exercise guidelines aimed at improving fitness in this population.
