# Corrigendum

**DOI:** 10.14814/phy2.12680

**Published:** 2016-01-05

In Liem et al. 2015, the following errors were published in the Results, Discussion sections:

In the Results (paragraph 7) and Discussion (paragraph 5) sections, “higher age” and “higher BMI” should have been “lower age” and “lower BMI”.

In paragraph 5 of the Discussion section, the following statement, “However, the inverse relationship between age and lower peak VO2 in our participants with SCA may reflect the cumulative impact of disease‐related factors on fitness with age.” should have been inserted after the statement that currently reads, “The developmental aspects of peak VO2 are also known to track with physical growth, pubertal maturation and increases in skeletal mass with age (Davies et al. 1972; Krahenbuhl et al. 1985; Armstrong et al. 1991).”

The authors apologise for the errors.
